# Creation of a Composite Bioactive Coating with Antibacterial Effect Promising for Bone Implantation

**DOI:** 10.3390/molecules28031416

**Published:** 2023-02-02

**Authors:** Elena G. Zemtsova, Lada A. Kozlova, Natalia M. Yudintceva, Daria N. Sokolova, Andrey Yu. Arbenin, Alexandra N. Ponomareva, Petr M. Korusenko, Ludmila A. Kraeva, Elizaveta V. Rogacheva, Vladimir M. Smirnov

**Affiliations:** 1 Institute of Chemistry, St. Petersburg State University, 7/9 Universitetskaya nab, 199034 Saint Petersburg, Russia; 2 Institute of Cytology, Russian Academy of Sciences (RAS), Tikhoretsky pr 4, 194064 Saint Petersburg, Russia; 3 Pasteur Institute of Epidemiology and Microbiology, 14 Mira Street, 197101 Saint Petersburg, Russia

**Keywords:** implant, template, electrochemical synthesis, silver (Ag), titanium (Ti), titanium dioxide (TiO_2_), hydroxyapatite (HAp), TiO_2_/Ag/HAp composite, bioactive coating

## Abstract

When creating titanium-containing bone implants, the bioactive coatings that promote their rapid engraftment are important. The engraftment rate of titanium implants with bone tissue depends significantly on the modification of the implant surface. It is achieved by changing either the relief or the chemical composition of the surface layer, as well as a combination of these two factors. In this work, we studied the creation of composite coatings with a two-level (the micro- and nanolevel) hierarchy of the surface relief, which have bioactive and bactericidal properties, which are promising for bone implantation. Using the developed non-lithographic template electrochemical synthesis, a composite coating on titanium with a controlled surface structure was created based on an island-type TiO_2_ film, silver and hydroxyapatite (HAp). This TiO_2_/Ag/HAp composite coating has a developed surface relief at the micro- and nanolevels and has a significant cytological response and the ability to accelerate osteosynthesis, and also has an antibacterial effect. Thus, the developed biomaterial is suitable for production of dental and orthopedic implants with improved biomedical properties.

## 1. Introduction

The duration of the recovery of a damaged bone after implant placement affects the quality of the patient’s life. Therefore, the implant engraftment rate is one of the key factors in implantation. This could be improved by the high biological activity of its coating, determined mainly by the chemical composition and topography of its surface.

Usually, implants are metal-based, e.g., alloys of titanium, cobalt, etc., which is due to their high mechanical properties and economic profitability. However, some implants may be rejected due to susceptibility to corrosion, susceptibility to infection, low biological activity and, consequently, inability to effectively integrate into the hostile environment of the body. This means that new bioactive systems for implants are needed [1,2]. Bioactive coatings for metal implants are relevant, since local therapeutic interventions are usually more desirable than systemic therapy because of their better bioavailability, which promotes rapid bone healing [3].

At the same time, it is well known from the literature data and clinical practice that there is a relationship between the morphology of the implant surface, biocompatibility and osseointegration [4,5,6]. It should be noted that the bioactive characteristics of the implant are influenced not only by the chemical composition of its coating, but also by the relief of the surface.

The presence of a micro-rough surface on the implant and nanoscale irregularities that mimic natural bone structure provide improved implant osseointegration [7,8], and, consequently, accelerate the bone’s healing.

The two-level hierarchy of the coating relief (roughness both in the micron and nanometer range) triggers the process of osteoblast differentiation and improves adhesion, respectively, increasing its material bioactivity [5,6,9,10].

It is a non-trivial task to obtain coatings with a two-level hierarchy. A promising strategy for the production of implants with biologically active hierarchical coatings, consisting of inorganic bioactive materials, includes a combination of several technological methods (sol-gel technology, etching, laser technologies) [11].

The implant engraftment is slowed by unwanted infection of the implant area [12]. Antibiotics could lead to bacterial resistance to drugs; therefore, various bactericidal (organic or inorganic) coatings are suitable. For implantation, such systems must be not only antibacterial, but also biocompatible in order to maintain the reproduction and functionality of surrounding tissues [13].

The usual inorganic antibacterial substances are metals and metal oxides, for example Ag (silver), Cu (copper), Au (gold) and Zn (zinc), in their micro- or nanoforms [13,14].

Ag is the most beneficial antibacterial substance due to its broad spectrum of bactericidal properties, as well as a high level of biocompatibility and stability [15,16]. Its high antifungal and antiseptic abilities are known. Despite the excellent antibacterial properties of silver, there are serious problems and controversies regarding the use of silver for medical implants. On the one hand, a solid silver coating has a negative effect on the viability, proliferation and early adhesion of osteoblast cells. The paper [17] describes the negative effect of a solid silver layer (1.29 wt.% or more) on the implant surface for early adhesion of osteoblast cells, mainly due to the toxicity of Ag ions. On the other hand, the osseointegration rate of the implant increases significantly due to the antibacterial effect of silver [18,19,20,21,22].

To increase the speed and improve the quality of implant engraftment, the implant surface is often modified with hydroxyapatite Ca_10_(PO_4_)_6_(OH)_2_ (HAp) or amorphous calcium phosphate, because HAp is one of the main components of the mineral part of the bones [23,24,25]. Composite coatings based on silver and HAp have good biocompatibility and antibacterial properties [26,27,28]. However, the published methods do not allow for the obtaining of the desired surface with a complex micro- and multi-level structure [29].

In our previous work [30], we showed the possibility of depositing island-type hydroxyapatite on the surface of a TiO_2_/Ag sample using pulsed electrochemical deposition, and we carried out preliminary comparative cytological studies. As a result, it has been shown that electrochemical deposition is not cytotoxic for osteoblast-like cells.

In this work, we continued the study begun in [30] and created an optimal TiO_2_/Ag/HAp composite coating with controlled composition and structure at the micro- and nanolevels using a combination of sol-gel synthesis and electrochemical deposition from a solution.

This bioactive island-type coating at the micron level improves the adhesion of osteoblast cells on the surface of the Ti implant and eliminates the negative effect of Ag in the TiO_2_ coating on the early adhesion of osteoblast cells.

The prospects of using this coating, which has antibacterial and high bioactive properties, for bone implantation have also been investigated.

## 2. Results and Discussions

### 2.1. Preparation of Composite Coating TiO_2_/Ag/HAp on Titanium

During the deposition of the TiO_2_ template, post-synthetic heat treatment temperatures between 150 °C and 500 °C in 50 °C increments were tested. The optimal temperature for creating a perforated (insular) oxide coating is 400 °C. At this temperature, the perforation areas are commensurate with the osteoblast cells, which positively affects the rate of the osteoblast cell monolayer formation on the implant surface.

**Figure 1:**
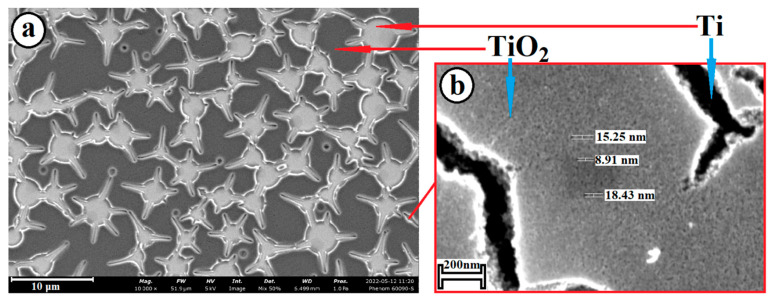
SEM images of TiO_2_ film: (**a**) with micro-roughness; and (**b**) with nanopores.

The structures were analyzed using SEM. Figure 1 shows a micrograph of a synthesized TiO_2_ film with the presence of micro- and nano-roughnesses. The two-level hierarchy, as noted above, significantly increases the bioactivity of the implant surface.

**Figure 2:**
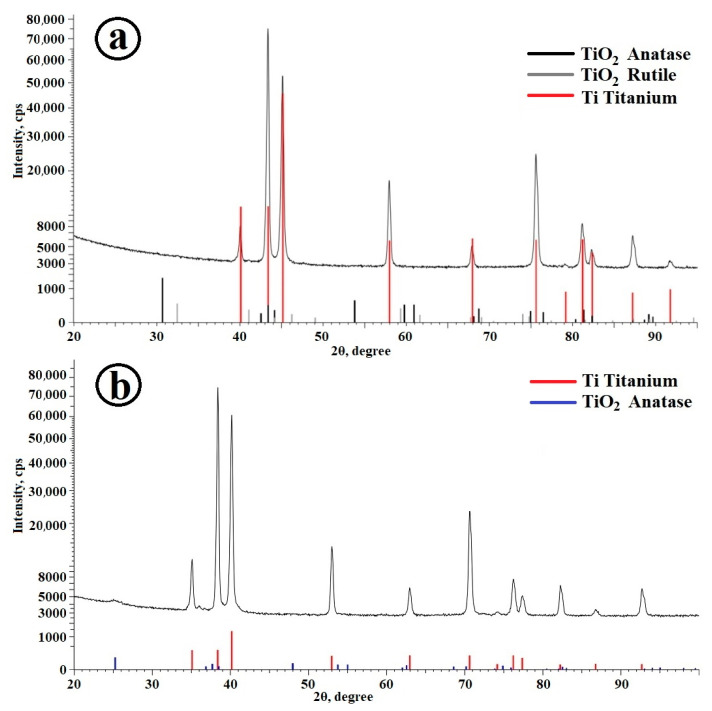
XRD patterns of the sample with a TiO_2_ coating: (**a**) before calcination; and (**b**) after calcination at 400 °C.

There are no anatase and rutile reflexes on the diffractogram of the resulting TiO_2_ coating (Figure 2a), which indicates the X-ray amorphousness of the deposited film. However, after 1 h calcination of a TiO_2_-coated sample at 400 °C, the film becomes polycrystalline. Anatase reflexes (Figure 2b) confirm the presence of the TiO_2_ phase in the sample.

At the second stage, a TiO_2_/Ag composite coating was obtained by electrochemical template synthesis using a TiO_2_ coating with insular perforations as a template.

Ag coatings can be synthesized on the implant surface by various methods, such as electroforming, vapor deposition, layer-by-layer deposition, vacuum plasma spraying, anodizing, electrochemical deposition [18,19,20,21,22,31], etc. In our work, the method of pulsed electrochemical deposition was used for Ag deposition. This method makes it possible to synthesize coatings with a uniform Ag particle distribution, high adhesion to the Ti surface and the ability to create an island-type coating.

**Figure 3:**
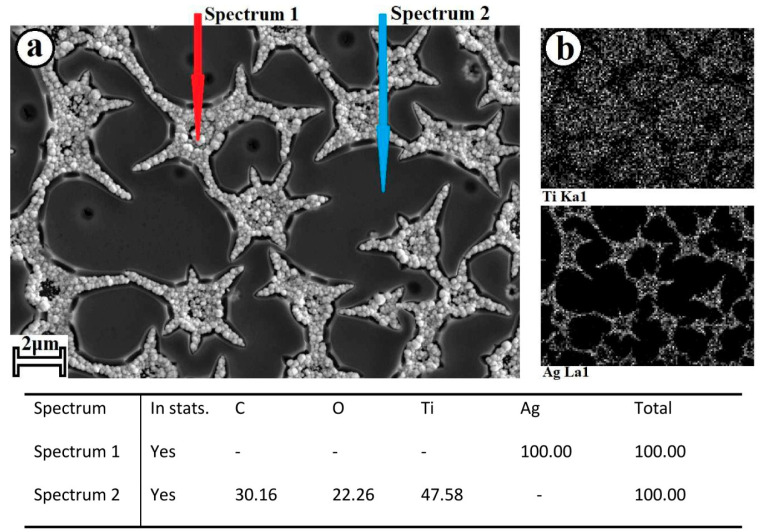
SEM image of (**a**) the TiO_2_/Ag composite; and (**b**) the element maps of the Ti and Ag in composite.

When using pulsed current in template electrochemical synthesis, Ag is deposited on a Ti substrate, repeating the geometry of island perforations formed by the TiO_2_ film. Figure 3 shows the SEM micrograph.

The Ag deposit clearly reproduces the structure of the TiO_2_ coating. Cracks emanating from micron pores are also filled with Ag particles. Also, the TiO_2_/Ag composite was studied by X-ray spectral microanalysis (EDX) with an electron probe using an X-ray microanalysis attachment on a scanning electron microscope. It can be seen from the Ti and Ag element maps (Figure 3b) that Ag is mainly deposited in the perforation of the TiO_2_ film, repeating its geometry. This indicates the successful implementation of the template electrochemical synthesis of the Ag coating.

**Figure 4:**
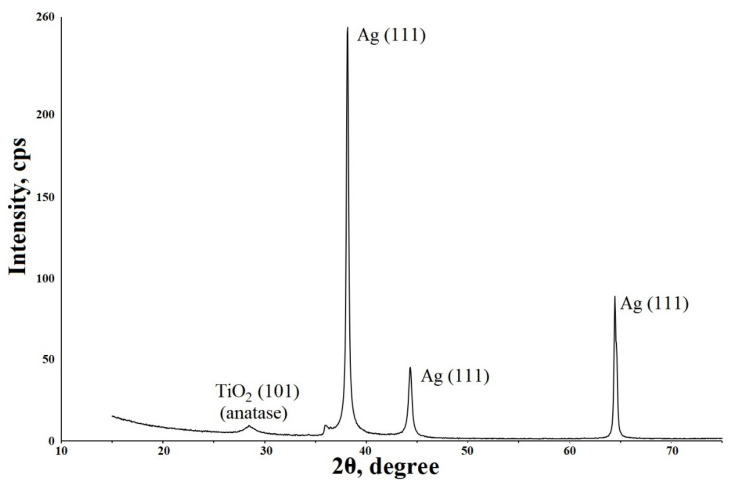
XRD patterns of the sample coated with TiO_2_/Ag.

The presence of silver is also confirmed by X-ray phase analysis data (Figure 4).

At the third stage, an island-type TiO_2_/Ag/HAp composite coating was obtained by template electrochemical deposition of HAp on the composite TiO_2_/Ag template coating obtained at the second stage. Pulsed deposition provided the structure of an intermittent HAp layer on the sample surface and made it possible to obtain an island-type coating convenient for the adhesion of osteoblast bone cells.

**Figure 5:**
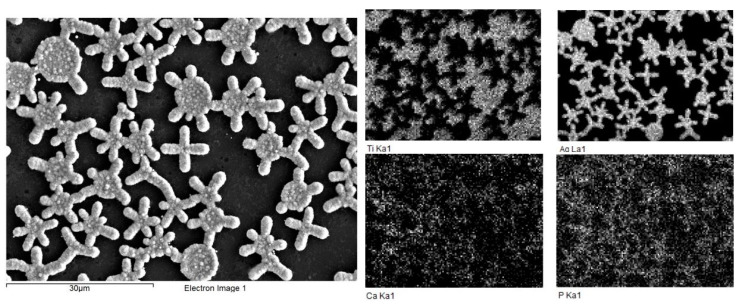
SEM image and element maps of Ti, Ag, Ca, P for TiO_2_/Ag/HAp composite.

Micrographs of the samples were obtained using SEM, and their element maps were compiled by X-ray spectral microanalysis with an electron probe (Figure 5).

**Figure 6:**
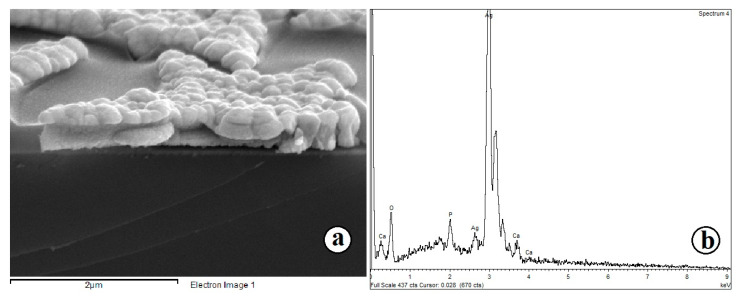
(**a**) SEM image of the chip of the sample with a HAp microparticle array obtained with template electrochemical synthesis; and (**b**) the EDX spectrum recorded at the corresponding points sample with a HAp.

Micrographs and element maps show that a high level of localization of calcium and phosphorus has been achieved, and the deposited HAp repeats the geometry of the TiO_2_/Ag template. Microscopy of the sample section showed that the resulting coating had a thickness of several hundred nanometers, and the HAp particles themselves rise above the surface of titanium, creating a micron roughness of the coating (Figure 6). This coating is also chemically close to the mineral composition of bone, which is important for bone implantation.

**Figure 7:**
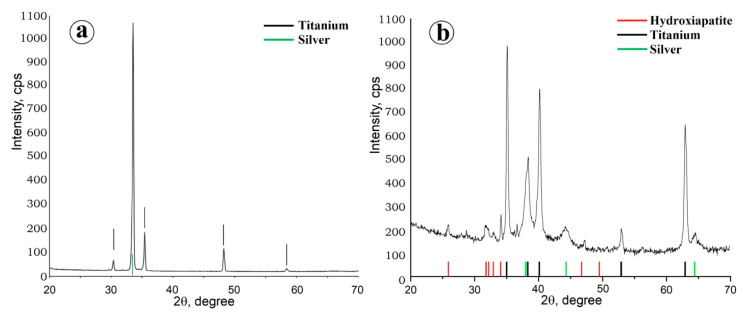
XRD patterns of the composite coating TiO_2_/Ag/HAp.

XRD confirms the crystal structure of the synthesized composite coating. The diffractogram (Figure 7) shows peaks of Ti substrate, silver (Ag microparticles forming the template structure) and HAp.

### 2.2. Cytological Studies of Samples

Proliferative activity (cytotoxicity) and adhesive properties of MG-63 osteoblast cells cultured on the surface of samples (Table 1) responsible for the formation of a cell monolayer on experimental samples was evaluated at the Institute of Cytology of the Russian Academy of Sciences (St. Petersburg).

#### 2.2.1. MTT Test (Cytotoxicity Assessment)

**Figure 8:**
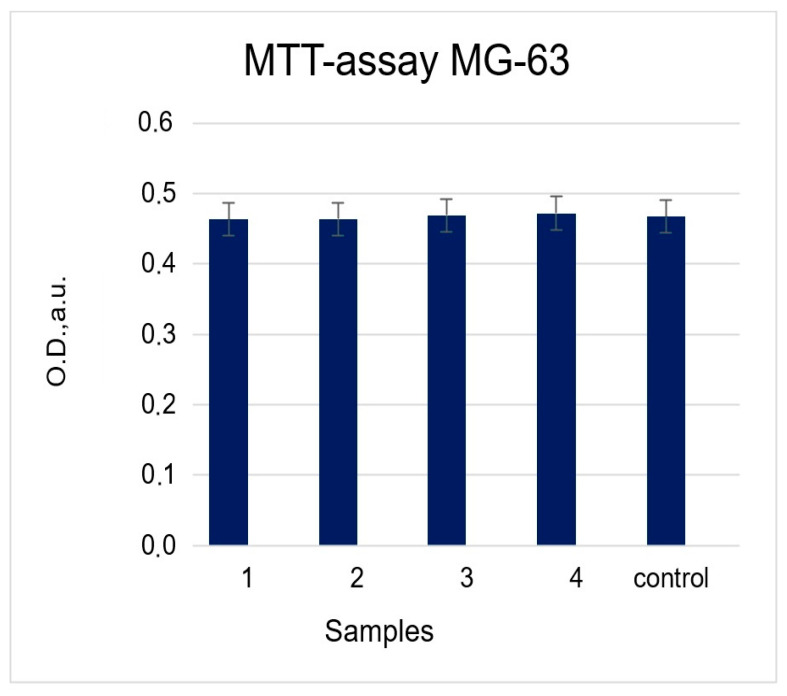
Proliferation activity of MG-63 cells on the samples.

The results of the evaluation of the proliferative activity of MG-63 cells cultured on the surface of the samples are shown in Figure 8. Based on these data, it is shown that when cells were grown on the surface of all the samples, their viability did not differ from the control during the entire period of joint incubation (48 h). The conclusion is made about the absence of cytotoxicity in the obtained composite coatings.

**Figure 9:**
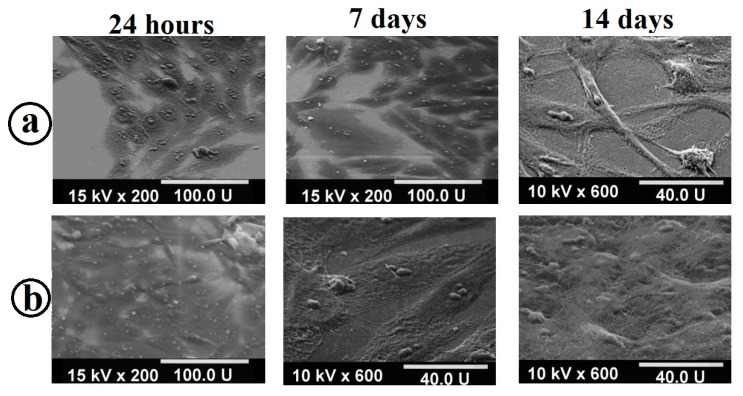
SEM images for the samples after adhesion and spreading of the human MG-63 cell line: (**a**) on polished titanium; and (**b**) on the sample with the TiO_2_/Ag/HAp coating.

Based on the obtained microphotographs of samples with osteoblast cells on the surface (Figure 9), it is concluded that a sample with a composite coating is characterized not only by high adhesive properties of osteoblasts, but also by the ability to develop a continuous cell layer, which indicates the possibility of accelerated osteosynthesis on such a surface. This is due to the presence of a two-level composite coating structure formed by micron islands of hydroxyapatite and nanometer pores of TiO_2_ xerogel for better adhesion of osteoblast cells. In addition, based on micrographs, it can be concluded that the resulting composite coating has no cytotoxicity and has the capacity for of more than 85% in 24 h for MG-63 osteoblast cells to adhere to it.

Cell differentiation in the osteogenic direction was studied using markers of early (alkaline phosphatase—ALP) and late (osteopontin—OPN) differentiation [24,32].

#### 2.2.2. Estimation of Alkaline Phosphatase (ALP) Content

**Figure 10:**
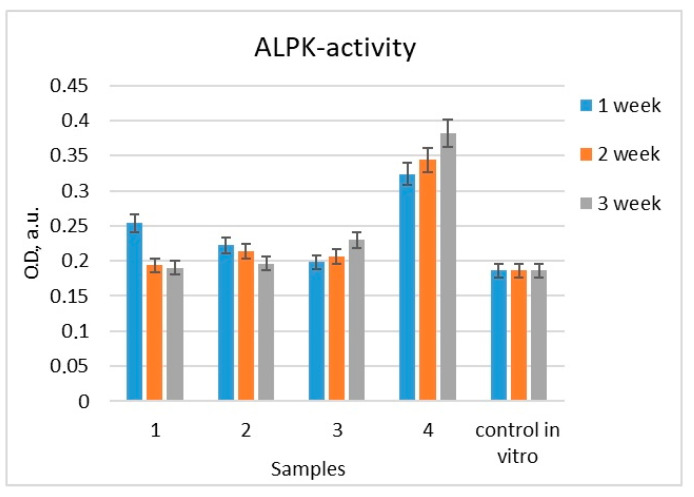
Changes in the level of alkaline phosphatase activity during differentiation of the MG-63 line in the osteogenic direction. (*Note:* Control in vitro is the cultivation of cells on the surface of plastic. The level of ALP activity in the medium from control cells was taken as 100% [cultivation on plastic].)

During three weeks of differentiation of MG-63 cells cultured on the surface of samples (no. 1–4), the level of ALP activity in the conditioned medium was higher compared to the control values (cells culture under standard conditions) (Figure 10).

At the same time, a gradual increase in the level of ALP activity was observed during cell differentiation on the surface of two samples (no. 3 and 4), where ALP activity increased by 24 and 105%, respectively. The level of ALP activity in the medium obtained from control cells (culturing on plastic) was taken as 100%. When cells were cultured on the surface of samples no. 1 and 2, the level of ALP activity gradually decreased. For example, after the first week of cell differentiation on the surface of the sample no. 1, the values of the ALP activity level were 34% higher compared to the control, and then decreased and no longer had significant differences with the control (Table 1).

Thus, during the differentiation of MG-63 cells on the surface of the samples, there was an increase in the level of ALP activity compared to the control (cell culture on plastic), which indicated the influence of the nature of the surface treatment of the material on the ability of MG-63 cells to differentiate in the osteogenic direction. At the same time, the treatment (TiO_2_/Ag, TiO_2_/Ag/HAp coatings) of the surface of samples no. 3 and 4 had the most significant effect on the differentiation of the cell line.

#### 2.2.3. Estimation of Osteopontin (OPN) Content

**Figure 11:**
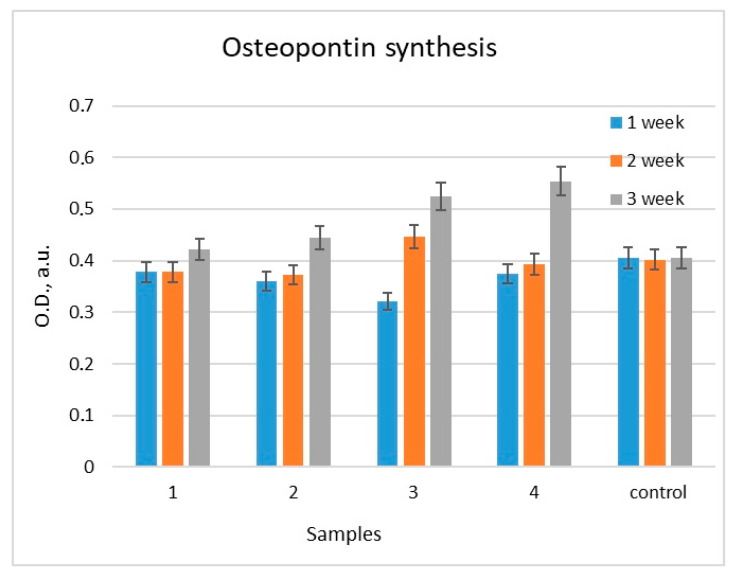
The content of osteopontin in the medium conditioned by MG-63 cells during differentiation in the osteogenic direction. (Note: Control is the cultivation of cells on the surface of plastic. The level of osteopontin in the medium from control cells was taken as 100% [cultivation on plastic].)

During three weeks of differentiation of MG-63 cells cultured on the surface of samples (no. 1–4), a gradual increase in OPN content was observed (Figure 11). The OPN concentration in the medium increased by 29 and 36% compared to the control after cell culture on the surface of samples no. 3 and 4, respectively. The OPN content in the medium obtained from control cells (culturing on plastic) was taken as 100%. When culturing cells on the surface of samples no. 1 and 2, no significant differences in the OPN content compared to the control were revealed.

In a series of in vitro experiments, we have shown that coatings with a complex hierarchical structure at the micro- and nanoscale and the composition of TiO_2_/Ag/HAp significantly enhanced the differentiation of osteoblasts in the osteogenic direction (Figure 10 and Figure 11) and contribute to improved implant engraftment to the bone.

### 2.3. The Antibacterial Activity of Samples with a Composite Coating TiO_2_/Ag/HAp

The study was carried out according to ISO Standard 22196 “Measurement of antibacterial activity on the surface of plastics and other non-porous materials.”

Microorganism suspensions were prepared at 10^7^ CFU/mL and 10^6^ CFU/mL according to turbidity standard. Control solutions were sown in the amount of 1 mL on nutrient media, and excess liquid was taken out with a dispenser. Samples were placed in Petri dishes. The samples were incubated in a thermostat at 37 °C for 24 h, then stored until the results were obtained in a refrigerator at 2–4 °C. On the composite coating TiO_2_/Ag/HAp, microorganism suspensions were planted in the amount of 200 mL. A similar procedure was done with control plates. The samples were covered with a sterile film Parafilm M 40 × 40 mm using tweezers and incubated in a thermostat with a closed lid at 37 °C for 24 h.

Next, the films were transferred from the test samples to separate sterile test tubes containing 9 mL of 0.9% NaCl, with an exposure time of 1 h. After exposure, 1 mL of physiological solution was sown on nutrient media, and the excess liquid was taken out with a dispenser. The samples were incubated in a thermostat at 37 °C for 24 h. Colonies were counted and the results of the experiment were taken into account.

As a result of the study of samples for antibacterial activity, the presence of antibacterial properties of samples containing silver structures in the form of islands was confirmed, compared with samples containing only a layer of titanium oxide (Table 2).

**Figure 12:**
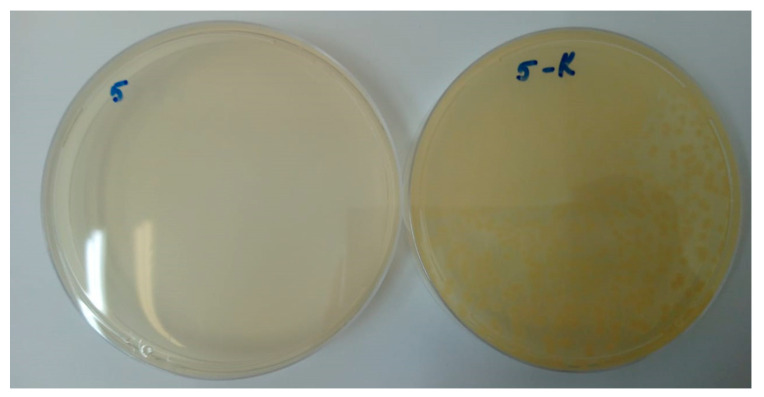
Culture of reference strain no. 7: *Pseudomonas aeruginosa* ATCC 27,853 on the sample with TiO_2_/Ag/HAp composite coating (**left**) and on a control sample (**right**).

It can be seen from the table that the growth of microorganisms was observed in the seeded control samples and in control samples without silver containing only the titanium oxide coating. In the composite coating of the island-type TiO_2_/Ag/HAp, there is a 100% absence of culture growth. In particular, Figure 12 shows a photograph illustrating a comparison of culture growth of the reference strain *Pseudomonas aeruginosa* ATCC 27,853 (no. 7) on the TiO_2_/Ag/HAp composite sample and the control sample. It can be seen that in the presence of Ag in the composition of the sample, there is a 100% absence of culture growth.

As a result of the study of samples for antibacterial activity, the presence of antibacterial properties of the samples was confirmed. The results of this study allow us to conclude that it is advisable to apply an insular composite silver coating on titanium implants.

The mechanisms of the antibacterial action of silver can be described in several ways [33]. One of them is based on the penetration of Ag^+^ ions into the peptidoglycan layer or bacterial wall, which leads to damage to DNA and bacterial proteins [34]. The formation of free radicals is also a possible pathway for the action of silver, since this destroys the cell membrane, changing its permeability, which ultimately leads to the death of the bacterium [15]. In addition, bacteria in their composition have sulfur and phosphorus, which are able to react with silver. Silver also disrupts the life cycle of bacteria [35].

Based on a large number of in vitro and in vivo studies and our experimental data, we came to the unequivocal opinion that for the fastest and most successful osseointegration of the implant, a developed relief is necessary both on the micro- and nanoscale scale [36,37,38]. Micron relief, and especially the presence of micropores, significantly improves the adhesion of bone tissue cells [38,39]. The presence of a specific nanorelief plays a significant role in improving circulation and accelerating the adsorption of biomolecules (proteins, nutrients) [38], and can also lead to the occurrence of an antibacterial effect [40,41]. To determine the success of the medical implant material, the composition of the surface is no less important a characteristic than the relief. Studies have shown that the composite coating of the TiO_2_/Ag/HAp composition with a developed surface relief at the micro- and nanoscale has a significant cytological response and the ability to accelerate osteosynthesis.

## 3. Materials and Methods

### 3.1. Preparation of TiO_2_/Ag/HAp Coating on Ti Surface

Firstly, TiO_2_ was coated on Ti as the template for further operations [32]. A technical Ti (VT1-6) plate (10 × 38 mm) was used with achieved roughness of <0.01 microns. A solution of absolute isopropanol (iPA), titanium tetraisopropoxide (TTIP), diethanolamine (DEA) and polyethylene glycol (PEG, Mw = 20,000 D, Merck, Rahway, NJ, USA) in the 773/227/105/50 ratio was used for the synthesis of the film. The coating was obtained by the sol-gel method using dip-coating technology using the equipment KSV Nima Dip Coater, Singlevessel. The extraction rate was 100 mm/min. Then the deposited film was heat treated at 400 °C.

Secondly, Ag was coated over the TiO_2_ film.

Silver was precipitated from an aqueous electrolyte containing AgNO_3_, sulfosalicylic acid, 25% aqueous ammonia to reach pH 9. Template deposition was carried out in a polypropylene cell with stirring, using a potentiostat-galvanostat Elins P45X. A Ti substrate coated with TiO_2_ was used as a working electrode, and silver was used as counter electrode.

The silver was deposited from the solution at pulsed current, since constant current gave insufficient repeatability of the template pattern without repeating the geometry of the perforations. Preliminarily, the optimal number of deposition cycles was determined. Each of 300 cycles included the impulse sequences 1 V—5 ms, 0.3 V—3 ms, 2 V—10 ms, 0 V—65 ms.

At the third stage, HAp structures were obtained on the surface of the TiO_2_/Ag composite coating as the template. As above, potentiostat-galvanostat Ellins P45X was used for electrochemical matrix synthesis (TiO_2_/Ag composite serves as cathode and matrix and graphite serves as anode). Based on HAp stoichiometry (Ca/P = 1.67), we used 1.18 g of Ca(NO_3_)_2_, (Vekton, Krasnodar, Russia) and 0.411 g of KH_2_PO_4_ (Vekton, Russia) per 100 mL of deionized water. HAp was deposited at pulsed current [30].

### 3.2. Evaluation of the Formation of a Cellular Monolayer of MC3T3-E1 Osteoblasts on Titanium Samples with Composite Coating TiO_2_/Ag/HAp

All samples were placed in Petri dishes and sterilized by ozonation. The suspension of the MS3T3-E1 cell line was applied in a small volume of nutrient medium (100 µL) in such a way that a “drop” formed on the surface of the sample. The concentration of cells during sowing was 1 × 10^5^/cm^2^ of the sample. Samples with a suspension of cells deposited on their surface in this way were placed in a CO_2_ incubator at a temperature of +37 °C for 3 h. After this time, during which cell adhesion on the surface of the samples is supposed to occur, a nutrient medium was added to the Petri dishes. As a control, the application of a suspension of cells on the surface of cultural dishes was used.

The assessment of the state of cells (the nature of adhesion and spreading of cells on the surface of the samples) was performed by the SEM method using a JSM-35.7 microscope (JEOL, Tokyo, Japan).

### 3.3. MTT Test (Cytotoxicity Assessment)

Titanium samples were sterilized under ozonation conditions, then incubated in 1 mL of nutrient medium for 24 h at 37 °C. Two samples were used for each variation. A suspension of MG-63 cells with the same concentration (5000 cells/well) was sifted into the wells of a 96-well board (Nunc, Waltham, MA, USA). The number of repetitions *n* = 5. After cell adhesion, the medium was changed to the medium obtained after the samples’ incubation. The cytotoxicity of the material on the cells was evaluated after 24 h using a Vibrant MTT Cell Proliferation Assay Kit (Life Technologies, Carlsbad, CA, USA) according to the manufacturer’s protocol.

### 3.4. Evaluation of Cell Differentiation in the Osteogenic Direction

Ti samples were sterilized using ozonation. The samples were placed in a Petri dish (Nunc, USA), and a suspension of MG-63 cells with a concentration of 1 × 10^5^ in 20 µL of culture medium DMEM/F12 containing 10% fetal cow serum and gentamicin (Gibco, Waltham, MA, USA) was applied to their surface (on the central part). After 2 h of incubation, the cells were adhered to the surface of the samples, then 180 µL of nutrient medium was added to each sample so that the cells on the surface of the samples were completely covered with the medium.

Cells were cultured on the surface of the samples in a CO_2_ incubator for 3 weeks with a change of medium once a week to a medium that induces differentiation (StemPro Osteogenesis Differentiation Kit, Thermofisher Scientific, Waltham, MA, USA). Standard conditions of cell culture on the surface of culture plastic were used as a control. At each change of medium, a cell-conditioned medium was sampled, which was placed at −80 °C for subsequent assessment of the presence of alkaline phosphatase (ALP) and the content of osteopontin in it using a Human ALP and SSP1 (Osteopontin) ELISA Kit in accordance with the manufacturer’s protocol. The measurements were carried out on a multimodal Thermo Scientific Varioskan LUX reader with a wavelength of 450 nm.

### 3.5. Studies of the Antibacterial Activity of the Surface of the TiO_2_/Ag/HAp Composite Coating

During the experiment, titanium plates covered with silver structures as well as pristine titanium samples were examined. The samples were not subjected to additional processing, including sterilization.

For the study, daily cultures of reference strains were used, in the amount of 10^6^ and 10^7^ CFU/mL: *Enterococcus faecalis ATCC 29812, Staphylococcus aureus ATCC 25912, Klebsiella pneumoniae ATCC 19882, Acinetobacter baumannii 987, Pseudomonas aeruginosa ATCC 27853*.

The controls were taken: (1) seeding of a suspension of microorganisms in the amount of 10^6^ CFU/mL on a Muller-Hinton agar for the reliability of the contamination of the suspension; and (2) Ti plates without Ag coating to confirm the negative antibacterial effect of samples on microorganisms in the absence of Ag.

To assess the presence/absence of microbial growth, the quantitative growth of bacteria on a dense nutrient medium was studied after sowing suspensions on agar from samples without silver after 24 h of incubation. The experiment was repeated twice, and the results are reproducible without errors.

This study was conducted according to ISO Standard 22196 “Measurement of antibacterial activity on the surface of plastics and other non-porous materials”.

### 3.6. Characterization of the Composite Coating TiO_2_/Ag/HAp

The structure and morphology of the coatings were studied using a Zeiss Merlin scanning electron microscope (Carl Zeiss Microscopy GmbH, Jena, Germany). For X-ray spectral microanalysis and the construction of element maps, we used the EDX console (Oxford Instruments INCAx-act) for a Zeiss Merlin scanning microscope. The MiniFlex II X-ray diffractometer (Rigaku Corporation, Japan) was used for X-ray phase analysis (λ = 1.5406 Å).

## 4. Conclusions

In the course of this work, a technique was developed for electrochemical template synthesis of a TiO_2_/Ag composite coating obtained by sequential application of the sol-gel method using dip-coating technology and electrochemical deposition. The resulting coating has a two-level orderliness of the relief, consisting of micro- and nano-irregularities, and due to this increase, the bioactivity of the implant, since it improves adhesion and promotes an accelerated process of osteoblast differentiation. It was also shown that the combined approach used to obtain coatings based on the sol-gel technology and electrochemical deposition method is not cytotoxic for osteoblast-like cells. The study of coatings containing a deposited layer of silver in the form of islands has shown that the preservation of the antibacterial effect of such coatings also leads to a reduction in the period of implant engraftment. Cytological studies of the obtained TiO_2_/Ag/HAp coatings have shown that coatings of this composition lead to accelerated osteosynthesis and are promising in the development of implantation products in dentistry and orthopedics.

## Figures and Tables

**Table 1:** Samples for cytological studies.

Sample 1	Sample 2	Sample 3	Sample 4
Polished VT 6 titanium	TiO_2_ on VT 6 titanium	TiO_2_/Ag on VT 6 titanium	TiO_2_/Ag/HAp on VT 6 titanium

**Table 2:** Studies of the antibacterial activity of TiO_2_/Ag/HAp composite coating.

Sample Composition				
Cultures of Strains	Control Muller—Hinton Agar	Control TiO_2_	TiO_2_/Ag Coating	TiO_2_/Ag/HAp Composite Coating
1. *Enterococcus faecalis 812* (10^7^ CFU/mL)	Microorganisms growth	Microorganisms growth	100% no growth	100% no growth
2. *Staphylococcus aureus* ATCC 25,912 (10^7^ CFU/mL)	Microorganisms growth	Microorganisms growth	100% no growth	100% no growth
3. *Klebsiella pneumoniae* ATCC 19,882 (10^7^ CFU/mL)	Microorganisms growth	Microorganisms growth	100% no growth	100% no growth
4. *Acinetobacter baumannii* 987 (10^6^ CFU/mL)	Microorganisms growth	Microorganisms growth	100% no growth	100% no growth
5. *Acinetobacter baumannii* 987 (10^7^ CFU/mL)	Microorganisms growth	Microorganisms growth	100% no growth	100% no growth
6. *Pseudomonas aeruginosa* ATCC 27,853 (10^6^ CFU/mL)	Microorganisms growth	Microorganisms growth	100% no growth	100% no growth
7. *Pseudomonas aeruginosa* ATCC 27,853 (10^7^ CFU/mL)	Microorganisms growth	Microorganisms growth	100% no growth	100% no growth

## Data Availability

Not applicable.

## Sample Availability

Samples of the compounds are not available from the authors.
